# Intensified immunosuppressive therapy in patients with immune checkpoint inhibitor-induced myocarditis

**DOI:** 10.1136/jitc-2020-001887

**Published:** 2020-12-08

**Authors:** Jennifer Cautela, Sarah Zeriouh, Melanie Gaubert, Laurent Bonello, Marc Laine, Michael Peyrol, Franck Paganelli, Nathalie Lalevee, Fabrice Barlesi, Franck Thuny

**Affiliations:** 1 University Mediterranean Centre of Cardio-Oncology (MEDI-CO centre), Unit of Heart Failure and Valvular Heart Diseases, Department of Cardiology, Nord Hospital, Centre for CardioVascular and Nutrition research (C2VN), INSERM 1263, INRAE 1260, Aix-Marseille University, Assistance Publique – Hôpitaux de Marseille, Marseille, Provence-Alpes-Côte d'Azur, France; 2 Mediterranean Group of Cardio-oncology (gMEDICO), Aix-Marseille-University, Marseille, Provence-Alpes-Côte d'Azur, France; 3 Technological Advances for Genomics and Clinics (TAGC), UMR/INSERM 1090, Aix-Marseille-University, Marseille, France; 4 Gustave Roussy, Drug Development Department (DITEP), Paris-Saclay University, Villejuif, France

**Keywords:** immunotherapy

## Abstract

**Background:**

Myocarditis is a rare but life-threatening adverse event of cancer treatments with immune checkpoint inhibitors (ICIs). Recent guidelines recommend the use of high doses of corticosteroids as a first-line treatment, followed by intensified immunosuppressive therapy (IIST) in the case of unfavorable evolution. However, this strategy is empirical, and no studies have specifically addressed this issue. Therefore, we aimed to investigate and compare the clinical course, management and outcome of ICI-induced myocarditis patients requiring or not requiring IIST.

**Methods:**

This case–control study included all patients consecutively admitted to The Mediterranean University Center of Cardio-Oncology (Aix-Marseille University, France) for the diagnosis of ICI-induced myocarditis according to Bonaca’s criteria and treated with or without IIST. In addition, we searched PubMed and included patients from previously published case reports treated with IIST in the analysis. The clinical, biological, imaging, treatment, all-cause death and cardiovascular death data of patients who required IIST were compared with those of patients who did not.

**Results:**

A total of 60 patients (69±12 years) were included (36 were treated with IIST and 24 were not). Patients requiring IIST were more likely to have received a combination of ICIs (39% vs 8%, p=0.01), and developed the first symptoms/signs of myocarditis earlier after the onset of ICI therapy (median, 18 days vs 60 days, p=0.002). They had a significantly higher prevalence of sustained ventricular arrhythmia, complete atrioventricular block, cardiogenic shock and troponin elevation. Moreover, they were more likely to have other immune-related adverse events simultaneously (p<0.0001), especially myositis (p=0.0002) and myasthenia gravis (p=0.009). Patients who required IIST were more likely to die from any cause (50% vs 21%, p=0.02). Among them, patients who received infliximab were more likely to die from cardiovascular causes (OR, 12.0; 95% CI 2.1 to 67.1; p=0.005).

**Conclusion:**

The need for IIST was more common in patients who developed myocarditis very early after the start of ICI therapy, as well as when hemodynamic/electrical instability or neuromuscular adverse events occurred. Treatment with infliximab might be associated with an increased risk of cardiovascular death.

## Background

Immune checkpoint inhibitors (ICIs) are monoclonal antibodies that restore the immune response of CD8+ and CD4+T cells against tumor tissue by blocking the inhibitory action of ligand/receptor interactions. They include programmed death-1 checkpoint inhibitor (PD-1_i_), PD ligand-1 checkpoint inhibitor (PD-L1_i_), cytotoxic T-lymphocyte-associated protein-4 inhibitor (CTLA-4_i_), and lymphocyte-activation gene 3 inhibitor (LAG-3 _i_).^1^ Although these drugs represent a major advance in the treatment of many cancers, they are associated with several immune-related adverse events (irAEs) that may lead to mitigated overall therapeutic efficacy.^2–4^ ICI-induced myocarditis is one of the most feared irAEs, as it is associated with a case fatality rate of approximately 40%.^5^ It exposes patients to a risk of acute heart failure and sudden death due to ventricular arrythmia, pulseless electrical activity or complete atrioventricular block.^6–11^ Histological studies have shown myocyte necrosis associated with CD4+ and CD8+T cell infiltration similar to that observed during acute cell rejection of transplanted hearts.^6 12^ Thus, recent American and European guidelines have recommended the discontinuation of ICIs, treatment with high doses of corticosteroids as first-line therapy, and intensified immunosuppressive therapy (IIST) as soon as evolution is unfavorable. It is then recommended to consider other immunosuppressive drugs, such as infliximab, mycophenolate mofetil (MMF), antithymocyte globulin (ATG) or tacrolimus.^13–15^ However, these guidelines are based on expert consensus without strong evidence, and no studies have analyzed these immunosuppressive therapeutic strategies. In addition, the use of other immunosuppressive therapies, such as abatacept, alemtuzumab, tocilizumab, intravenous Ig and plasma exchange, have been recently described in a few case reports.^16–18^

In an effort to provide more data on the utilization of IIST, we aimed to investigate and compare the clinical course, management, and outcome of ICI-induced myocarditis patients requiring or not requiring IIST in a case–control study.

## Methods

### Study design and participants

We conducted a retrospective case–control study. From March 1 2015 to March 1 2020, the medical records of consecutive patients with a clinical suspicion of ICI-induced myocarditis were reviewed from the databases of The University Mediterranean Center of Cardio-Oncology in the North Hospital (Aix-Marseille University, France), a referral teaching hospital. During this period, patients were referred to our center when physicians had suspected myocarditis on the basis of clinical, biological or imaging cardiovascular evidence. From January 2018, all patients receiving ICI therapy in our center were also followed according to a standardized protocol. It included a cardio-oncology clinical visit with an ECG, transthoracic echocardiogram (TTE), and ultrasensitive troponin measurement (I then T from January 2019) before the beginning of treatment. Then, troponin measurement and ECG were performed before each ICI administration. In the case of cardiovascular symptoms/signs, recent ECG changes, or troponin increase >99th percentile of the upper reference limit provided by the manufacturing companies, patients were referred to our center for workup. Additional investigations, including cardiovascular MR (CMR) or endomyocardial biopsy, were performed according to the physician’s decision. Myocarditis management was left to the physician’s discretion. Only patients with a diagnosis of definite, probable, or possible myocarditis according to Bonaca’s criteria^19^ were included in the study. Patients were divided into two groups based on whether they were receiving an IIST defined as immunosuppressive therapy other than corticosteroids. All patients had given their consent on admission to permit the use of their personal medical data for research purposes.

In an effort to increase the size of the IIST patient group, we also included patients from previously published case reports who were treated for ICI-induced myocarditis with IIST. To identify these cases, we searched PubMed for articles using all combinations between group A and group B search terms with a cut-off date of April 2020. The group A search terms included the following: “myocarditis”, “cardiotoxicity”, “heart failure”, “adverse events”, “cardiac”, “cardiovascular”, “heart”, “pericarditis”, “myocardium” and “myocardial”. The group B search terms included the following: “immunotherapy”, “immunotherapies”, “checkpoint blockade”, “checkpoint inhibitors”, “CTLA-4”, “PD-1”, “PD-L1” and “LAG-3”. This search was limited to English articles. We also searched the reference lists of articles identified by this search strategy and selected additional references that we judged to be relevant. We finally selected the cases reporting in detail the patient characteristics, the evolution of the disease, and IIST as defined above. We ensured that each patient was included only once. Among the selected cases, we excluded those for which data were considered insufficient to formally draw a conclusion on definite, probable, or possible ICI-induced myocarditis according to Bonaca’s criteria.^19^ The patients of our center and those previously published were confirmed by two independent researchers.

The Strengthening the Reporting of Observational Studies in Epidemiology statement was followed to ensure the quality of data reported in this study.

### Data collection

Data of interest were extracted retrospectively from electronic medical records and case report publications. These included standard demographic, cardiovascular risk factor, other irAEs, ECG, TTE, CMR and biomarker data. Myocarditis-CMR diagnosis was defined as the presence of two out of two 2018-Lake Louise major criteria,^20^ or one out of two criteria associated with a plausible clinical scenario. Positive troponin was defined as a serum level >99th percentile of the URL associated with a dynamic evolution. Cancer-specific covariates included the type of cancer, ICI therapy and prior cancer treatment. Myocarditis-specific covariates also included clinical presentation, physical examination, CMR data, biopsy data and myocarditis treatments. Myocarditis complications were analyzed, and each myocarditis episode was graded according to the guidelines of The American Society of Clinical Oncology.^13^ For patients admitted to our center, the diagnosis of immune-related neuromuscular disorders was made by experienced neurologists by integrating the results of their clinical evaluation, serum CK levels, the presence of autoantibodies, the results of electroneuromyography and the neuromuscular biopsy. For reported cases, this diagnosis was considered in the analysis if the authors reported it in their description.

### IIST and outcome

Indications for IIST were classified as (1) hemodynamic, defined as the development, persistence or aggravation of heart failure syndrome or decreased left ventricular ejection fraction despite corticosteroid therapy; (2) electrical, defined as the development, persistence, or aggravation of ventricular arrhythmia or severe cardiac conduction abnormality despite corticosteroid therapy; (3) biological, defined as no decrease or increase in troponin levels after more than 3 days of corticosteroid therapy; (4) other, defined as the presence of severe noncardiac irAEs or a decision by physicians to initiate IIST in the absence of the other previous indications.

Study outcomes were deaths from any cause and myocarditis-related cardiovascular deaths.

For patients included in our center, the vital status was determined within the year after the day of admission. For patients previously published in case reports, the time from admission to death was always specified in the publication. Therefore, we also considered only deaths occurring within the year after the day of admission. Since we did not have the date of the vital status for some patients who survived the myocarditis episode, we considered them to be alive at 1 year.

### Statistical analysis

Continuous variables are expressed as means±SDs or medians (IQR) and compared using an unpaired t-test or Mann-Whitney U test. Categorical variables are expressed as frequencies (percentages) and compared using the χ^2^ or Fisher exact test. ORs and 95% CIs were estimated by logistic regression. All tests of significance were two sided, and a p value of 0.05 was considered significant. Statistical analysis was performed using SPSS V.20.0 (SPSS).

## Results

### Study population

**Figure 1:**
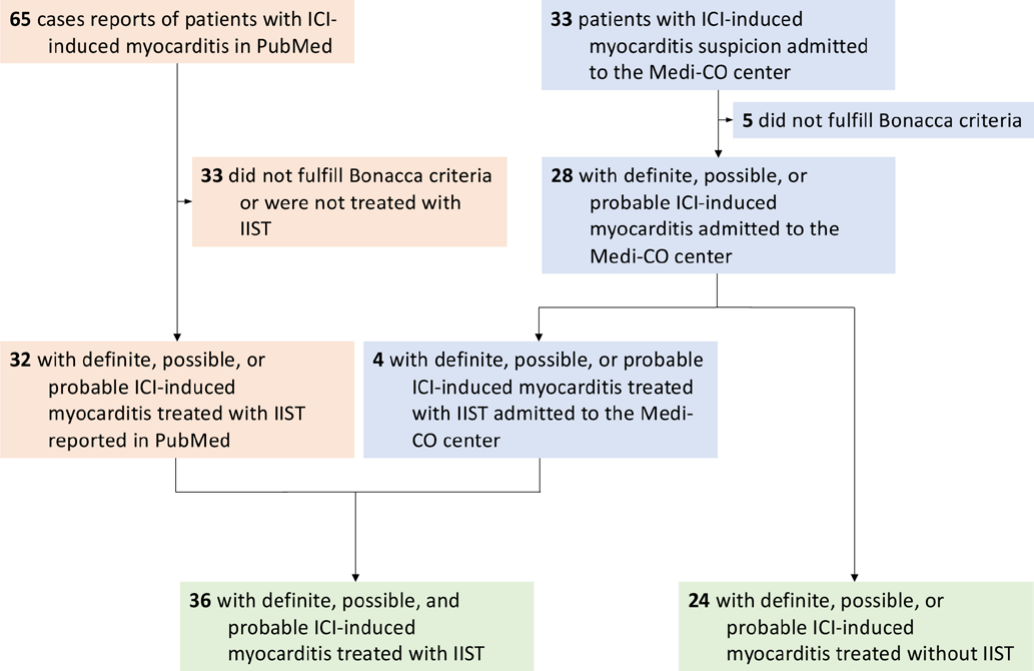
Study flow chart. ICI, immune checkpoint inhibitor; IIST, intensified immunosuppressive therapy.

**Figure 2:**
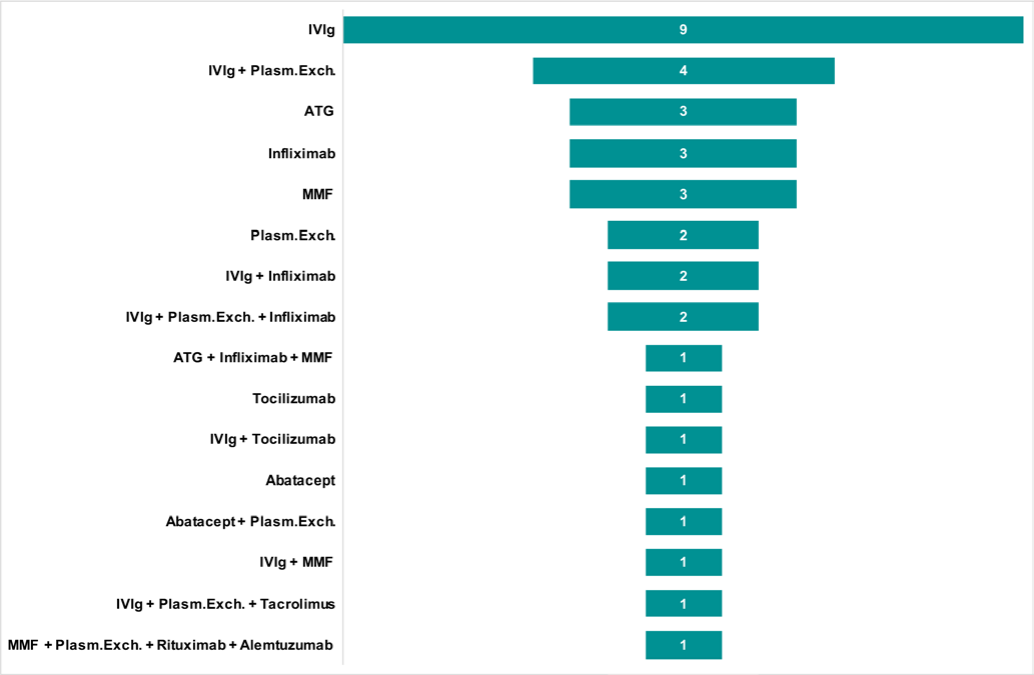
Intensified immunosuppressive therapy. ATG, antithymocyte globulin; ICI, immune checkpoint inhibitor; IIST, intensified immunosuppressive therapy; IV, intravenous; MMF, mycophenolate mofetil; Plasm.Exch., plasma exchange therapy.

From March 1 2015 to January 2020, a total of 33 consecutive patients in whom ICI-induced myocarditis was suspected were admitted to our center. Of these patients, five were excluded because they did not fulfill Bonaca’s criteria for the diagnosis of myocarditis. Of the 28 on-site patients, four (14%) required IIST. Additionally, our research identified 65 published case reports of ICI-induced myocarditis. Of them, 33 were excluded because they did not fulfill Bonaca’s criteria and/or were not treated with IIST (online supplemental table S1). A total of 60 patients (69±12 years) were finally analyzed in this study (36 were treated with IIST and 24 were not, figure 1). The type of IIST is shown in figure 2. It involved intravenous Ig (n=20), ATG (n=4), infliximab (n=8), tocilizumab (n=2), rituximab (n=1), MMF (n=6), abatacept (n=2), alemtuzumab (n=1), tacrolimus (n=1) and plasma exchange therapy (n=11). Combinations of these therapies were used in eight patients (22%). The reasons for IIST were hemodynamic (n=10), electrical (n=2), biological (n=7) and others (n=15).

10.1136/jitc-2020-001887.supp1 Supplementary data

### Baseline characteristics before myocarditis

The baseline characteristics of the two groups of patients are shown in table 1. In comparison with patients treated without IIST, IIST patients had a lower prevalence of smoking history and non-small-cell lung cancer. Other demographic characteristics and other types of cancer did not differ significantly between the two groups. Regarding immunotherapy, IIST patients were more likely to have received a combination of ICIs, especially PD-1_i_ with CTLA-4_i_ (39% vs 8%, p=0.01).

**Table 1:** Baseline characteristics

	IIST (n=36)	No IIST (n=24)	P value
Age, years	69±11	69±11	0.88
Female	16 (44)	9 (38)	0.59
CV risk factors and diseases			
Current or prior smoking*	4 (17)	11 (46)	0.03
Hypertension*	9 (38)	14 (22)	0.15
Diabetes mellitus*	6 (25)	2 (8)	0.25
Dyslipidemia*	5 (21)	2 (8)	0.42
Coronary artery disease*	2 (8)	3 (13)	1.0
Stroke*	1 (4)	0 (0)	1.0
Atrial fibrillation*	1 (4)	3 (13)	0.61
Heart failure*	0 (0)	2 (8)	0.49
COPD*	0 (0)	3 (13)	0.23
Cancer			
Melanoma	19 (53)	9 (38)	0.25
Non-small-cell lung cancer	6 (17)	11 (46)	0.01
Renal cell carcinoma	4 (11)	1 (4)	0.64
Gastric carcinoma	1 (3)	1 (4)	1.0
Glioblastoma	1 (3)	0 (0)	1.0
Myelodysplastic syndrome	2 (6)	0 (0)	0.51
Mesothelioma	1 (3)	0 (0)	1.0
Thymoma	1 (3)	0 (0)	1.0
Head and neck	0 (0)	2 (8)	0.16
Uterus	1 (3)	0 (0)	1.0
Prior chemotherapy†	15 (60)	15 (63)	0.86
Overall types of ICI			
Nivolumab (PD-1_i_)	23 (64)	14 (58)	0.67
Pembrolizumab (PD-1_i_)	9 (25)	2 (8)	0.17
Sintilimab (PD-1_i_)	1 (3)	0 (0)	1.0
Atezolizumab (PD-L1_i_)	0 (0)	4 (17)	0.02
Durvalumab (PD-L1_i_)	2 (6)	0 (0)	0.51
Ipilimumab (CTLA-4_i_)	16 (44)	4 (17)	0.03
Tremelimumab (CTLA-4_i_)	1 (3)	0 (0)	1.0
Relatlimab (LAG-3_i_)	0 (0)	3 (13)	0.06
Any PD-1_i_/PD-L1_i_	34 (94)	23 (96)	1.0
Any PD-1_i_	33 (92)	17 (71)	0.07
Any PD-L1_i_	1 (3)	6 (25)	0.009
Any CTLA-4_i_	17 (47)	4 (17)	0.03
Any LAG-3_i_	0 (0)	3 (13)	0.06
Single ICI agent			
Nivolumab	9 (25)	9 (38)	0.3
Pembrolizumab	7 (19)	2 (8)	0.29
Atezolizumab	0 (0)	4 (17)	0.02
Ipilimumab	2 (6)	1 (4)	1.0
Durvalumab	0 (0)	1 (4)	0.4
Sintilimab	1 (3)	0 (0)	1.0
Combination ICI			
Any PD-1_i_/PD-L1_i_ + CTLA-4_i_	15 (42)	3 (13)	0.01
Nivolumab+ipilimumab	14 (39)	2 (8)	0.01
Durvalumab+tremelimumab	1 (3)	0 (0)	1.0
Nivolumab+relatlimab	0 (0)	1 (4)	0.4
Durvalumab+tremelimumab	1 (3)	0 (0)	1.0
Nivolumab+ipilimumab+relatlimab	0 (0)	1 (4)	0.4

Values are mean±SD, n (%).

*Twenty-four of the 36 IIST patients had this information.

†Twenty-five of the 36 IIST patients had this information.

COPD, chronic obstructive pulmonary disease; CTLA-4_i_, cytotoxic T-lymphocyte-associated protein-4 inhibitor; CV, cardiovascular; ICI, immune checkpoint inhibitor; IIST, intensified immunosuppressive therapy; PD-1_i_/PD-L1_i_, programmed death-1 checkpoint inhibitor/programmed death-1 checkpoint inhibitor.

### Myocarditis presentation, clinical course and management

**Figure 3:**
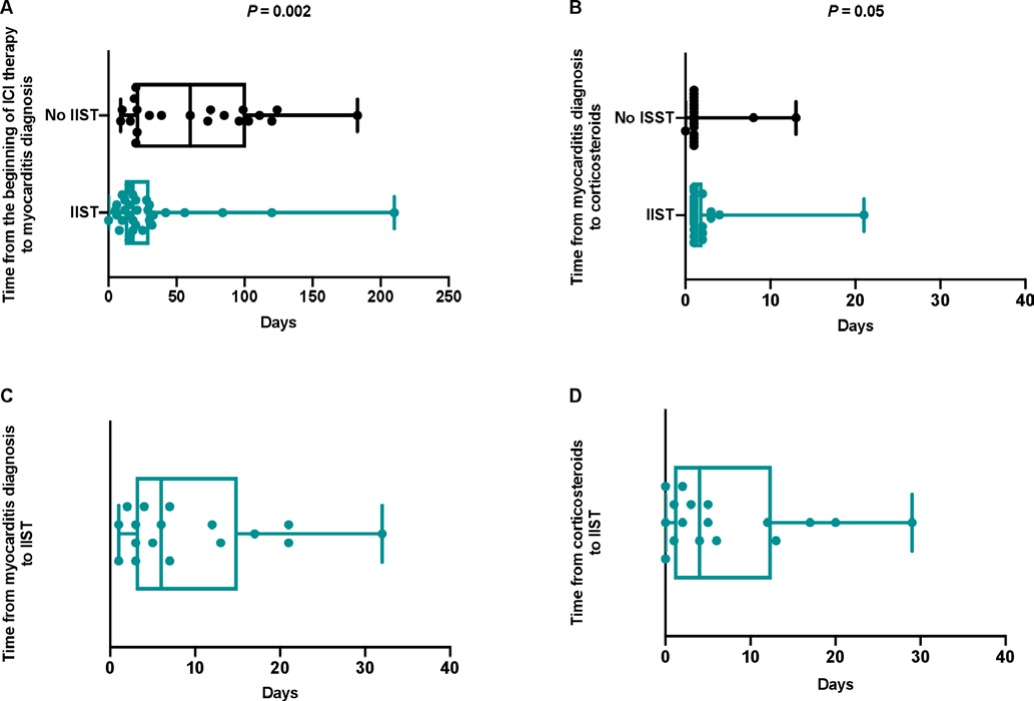
Times between beginning of immune checkpoint inhibitor therapy, onset of myocarditis, corticosteroid therapy and intensified immunosuppressive therapy. ICI, immune checkpoint inhibitor; IIST, intensified immunosuppressive therapy.

The median time from first ICI administration to the onset of myocarditis was 21 days (IQR 15–63 days). It was significantly shorter in patients who subsequently required IIST (18 days (IQR 12–30 days) vs 60 days (IQR 20–201 days), p=0.002) (figure 3). These patients were more likely to have a more severe form of myocarditis (table 2). Indeed, they had a significantly higher prevalence of sustained ventricular arrhythmia, complete atrioventricular block, cardiogenic shock and troponin elevation. Moreover, they were more likely to have other irAEs simultaneously (p<0.0001), especially myositis (p=0.0002) and myasthenia gravis (p=0.009).

**Table 2:** Myocarditis presentation and clinical course

	IIST (n=36)	No IIST (n=24)	P value
Time from beginning ICI to onset myocarditis, days*	18 (12–30)	60 (20–101)	0.002
Clinical presentation			
Chest pain	4 (11)	3 (13)	1.0
Shortness of breath	22 (61)	11 (46)	0.24
Palpitations	5 (14)	1 (4)	0.39
Pericardial effusion	1 (3)	0 (0)	1.0
Asymptomatic troponin elevation	7 (19)	8 (33)	0.22
ECG on admission			
Atrial fibrillation	8 (22)	3 (13)	0.50
Ventricular tachycardia	4 (11)	1 (4)	0.64
Complete atrioventricular block†	12 (34)	0 (0)	0.002
Complete left bundle branch block†	3 (9)	2 (8)	1.0
Complete right bundle branch block†	8 (23)	2 (8)	0.29
T wave or ST segment abnormality†	8 (23)	3 (13)	0.51
LVEF on admission, %‡	50±14	55±12	0.12
CMR			
Performed	20 (56)	20 (83)	0.03
Myocarditis-CMR diagnosis	18 (90)	19 (95)	1.0
Elevated troponin‡	32 (89)	17 (71)	0.02
Endomyocardial biopsy			
Performed	10 (28)	0 (0)	0.004
Positive for myocarditis	9 (90)	–	–
Myocarditis diagnosis			
Definite	25 (69)	14 (58)	0.35
Probable	6 (17)	3 (13)	
Possible	5 (14)	7 (29)	
Myocarditis grade§			
Grade 1	0 (0)	0 (0)	0.017
Grade 2	0 (0)	4 (17)	
Grade 3	0 (0)	1 (4)	
Grade 4	36 (100)	19 (79)	
Myocarditis-related complications			
Cardiogenic shock	7 (19)	0 (0)	0.03
Ventricular tachycardia or complete atrioventricular block	15 (42)	1 (4)	0.001
Sustained ventricular arrhythmia	8 (22)	1 (4)	0.04
Complete atrioventricular block	15 (42)	1 (4)	0.002
Other irAEs			
Any irAEs	29 (81)	6 (25)	<0.0001
Myositis	24 (67)	4 (17)	0.0002
Myasthenia gravis	12 (33)	1 (4)	0.009
Dermatitis	4 (11)	0 (0)	0.14
Thyroiditis	3 (8)	1 (4)	1.0
Polyradiculoneuritis	2 (6)	0 (0)	0.51
Arthritis	0 (0)	1 (4)	0.40
Uveitis	1 (3)	0 (0)	1.0
Corticosteroids	33 (92)	21 (88)	0.60
Time from first irAEs to corticosteroids, days¶	1 (1–2)	1 (1–1)	0.71
Initial supportive therapy			
Mechanical ventilation	12 (33)	2 (8)	0.03
Diuretics**	4 (13)	6 (25)	0.31
Beta-blockers‡	6 (19)	7 (29)	0.16
Angiotensin-converting enzyme inhibitors*	3 (9)	8 (33)	0.04
Inotropic agents or vasopressors	7 (19)	0 (0)	0.04
Mechanical assist device	3 (8)	0 (0)	0.27
Pacing	12 (33)	0 (0)	0.0008
Study outcomes			
Death from any cause	18 (50)	5 (21)	0.02
Death from cardiovascular cause	7 (19)	1 (4)	0.13

Values are mean±SD, median (IQR), or n (%).

*Thirty-three of the 36 IIST patients had this information.

†Thirty-five of the 36 IIST patients had this information.

‡Thirty-two of the 36 IIST patients had this information.

§According to the ASCO guidelines.^13^

¶Seventeen of the 36 IIST patients had this information.

**Thirty of the 36 IIST patients had this information.

ASCO, American Society of Clinical Oncology; CMR, cardiovascular MR; ICI, immune checkpoint inhibitor; IIST, intensified immunosuppressive therapy; irAEs, immune-related adverse events; LVEF, left ventricular ejection fraction.

Corticosteroids were used in 90% of patients without a significant difference in the timing of administration between the two groups. The median times from the onset of myocarditis to IIST and from the onset of corticosteroids to IIST were 6 days (IQR 3–15 days) and 4 days (IQR 1–13 days), respectively (figure 3).

### Study outcomes

**Figure 4:**
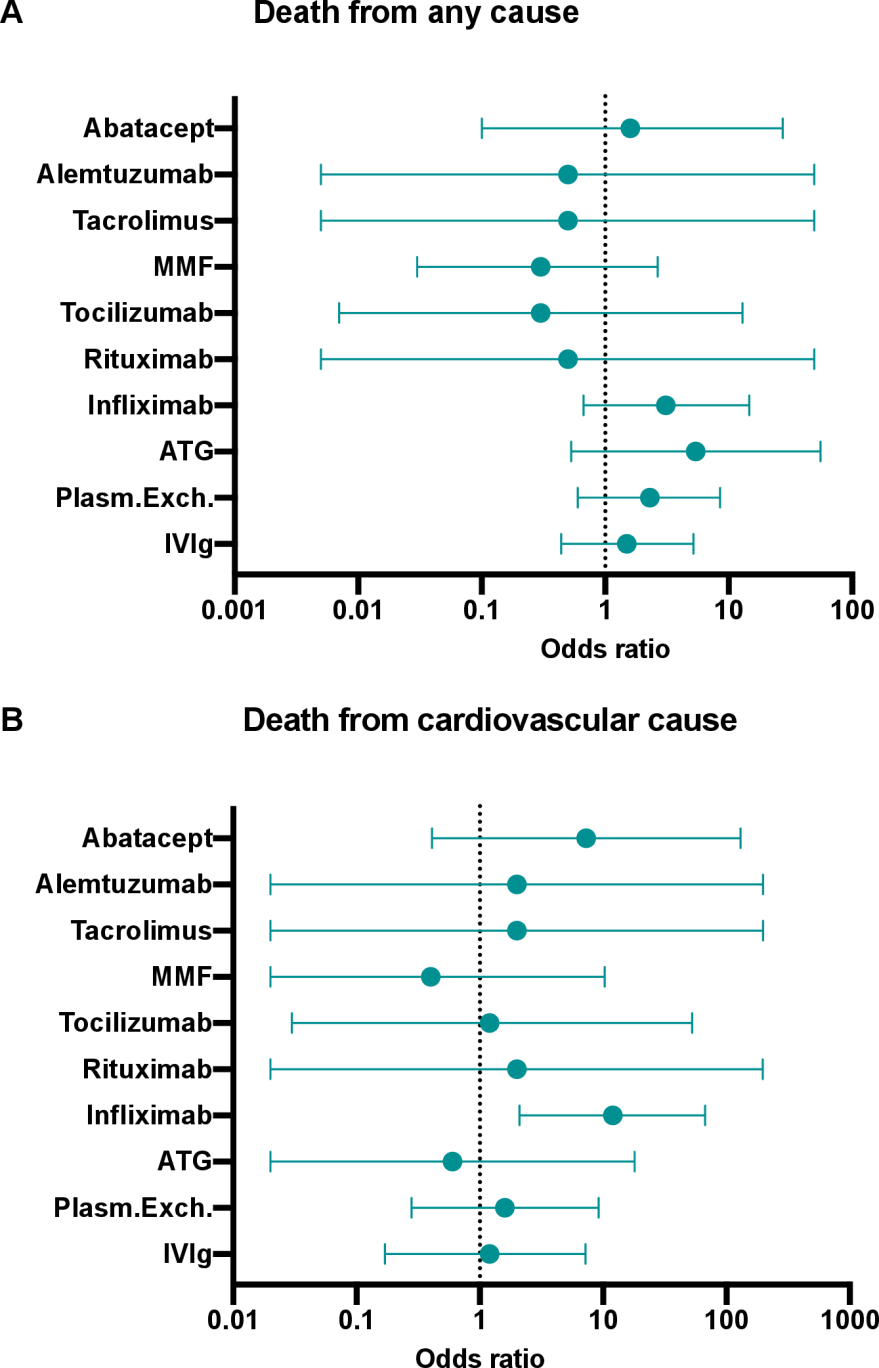
Forest plot of the risk of death from any cause (A) and from a cardiovascular cause according to the type of IIST. ATG, antithymocyte globulin; IIST, intensified immunosuppressive therapy; IV, intravenous; MMF, mycophenolate mofetil; Plasm.Exch., plasma exchange therapy.

Twenty-three patients (38%) died from any cause, and eight (13%) died from cardiovascular causes. Patients who required IIST were more likely to die from any cause (50% vs 21%, p=0.02) (table 2). According to the type of IIST, patients who received infliximab were more likely to die from cardiovascular causes (OR 12.0; 95% CI 2.1 to 67.1; p=0.005) (figure 4) (table 3). Indications for infliximab as a single or combination therapy were hemodynamic (n=4), biological (n=1) and others (n=3).

**Table 3:** All-cause and cardiovascular mortality according to intensified immunosuppressive therapy

	All-cause mortality	OR (95% CI)	P value	Cardiovascular mortality	OR (95% CI)	P value
No IIST	5/24 (21)	0.3 (0.06 to 0.96)	0.02	1/24 (4)	0.2 (0.004 to 1.60)	0.13
IVIg	9/20 (45)	1.5 (0.44 to 5.18)	0.57	3/20 (15)	1.2 (0.17 to 7.22)	1.0
Plasm.Exch.	6/11 (55)	2.3 (0.60 to 8.50)	0.23	2/11 (18)	1.6 (0.28 to 9.20)	0.60
ATG	3/4 (75)	5.4 (0.53 to 55.4)	0.16	0/4 (0)	0.6 (0.02 to 18.04)	0.79
Infliximab	5/8 (63)	3.1 (0.67 to 14.7)	0.15	4/8 (50)	12.0 (2.1 to 67.1)	0.005
Rituximab	0/1 (0)	0.5 (0.005 to 49.3)	0.78	0/1 (0)	2.0 (0.02 to 197.1)	0.76
Tocilizumab	0/2 (0)	0.3 (0.007 to 12.92)	0.52	0/2 (0)	1.2 (0.03 to 52.62)	0.93
MMF	1/6 (17)	0.3 (0.03 to 2.66)	0.27	0/6 (0)	0.4 (0.02 to 10.3)	0.59
Tacrolimus	0/1 (0)	0.5 (0.005 to 49.3)	0.78	0/1 (0)	2.0 (0.02 to 197.9)	0.76
Alemtuzumab	0/1 (0)	0.5 (0.005 to 49.3)	0.78	0/1 (0)	2.0 (0.02 to 197.9)	0.76
Abatacept	1/2 (50)	1.6 (0.10 to 27.5)	0.73	1/2 (50)	7.3 (0.41 to 130.1)	0.18

Values are n (%).

ATG, antithymocyte glogulin; IIST, intensified immunosuppressive therapy; IV, intravenous; MMF, mycophenolate mofetil; Plasm.Exch., plasma exchange therapy.

## Discussion

The management of irAEs during ICI therapy is challenging because some of them can lead to life-threatening complications.^2 6–9 21^ Despite many limitations, our study is the first to investigate the treatment of ICI-induced myocarditis by immunosuppressive therapy other than corticosteroids alone. In our opinion, it provides two important results. The first is to have clarified the profile of patients who required the intensification of their immunosuppressive treatment. Compared with patients not treated with IIST, patients who required IIST were more likely to receive a combination of ICIs and to experience myocarditis earlier after the first ICI administration. Moreover, the episode of myocarditis was more frequently complicated by hemodynamic or electrical instability and associated with neuromuscular irAEs. The second important finding was that infliximab was associated with a greater risk of cardiovascular death in patients requiring IIST. To the best of our knowledge, this is the first study that raises concern over infliximab use as second-line therapy.

Although any organ can be involved in ICI therapy, some irAEs may be very serious and lethal, such as myocarditis. This adverse event is infrequent but is associated with a high case fatality rate.^13 14^ It most commonly occurs with combined ICI therapy (especially PD-1i/PD-L1i+CTLA-4i) within the first months after the initiation of cancer treatment.^8 11^ The high risk of death justifies monitoring and management strategies for which strong data are lacking to provide recommendations with a high level of evidence. However, in the recent American and European guidelines, experts highlight the importance of very early diagnosis and management.^13 14^ As soon as a diagnosis of ICI-induced myocarditis is suspected, ICI treatment should be interrupted, the patient should be admitted to a cardiology unit (ideally in the intensive care unit), and corticosteroid treatment should be promptly initiated.^22^ Therefore, it is of crucial importance to detect patients with more severe myocarditis for whom corticosteroid therapy will be insufficient and IIST will be needed. From this perspective, our study provides insights into the profile of patients requiring IIST who will, therefore, be closely monitored. These were those presenting the first signs of myocarditis very early after the start of ICI therapy. While previous studies have shown that the median time was approximately 30 days for all patients with ICI-induced myocarditis,^11^ in our work, it was only 18 days for patients who were going to require IIST vs 60 days for others. Therefore, early onset of myocarditis symptoms/signs after the start of ICI treatment should encourage clinicians to be more attentive to the evolution of these patients, especially if they had received a combination of ICIs. The analysis of ICI-induced myocarditis cases based on the need for IIST allows us to confirm that the occurrence of cardiogenic shock, ventricular arrhythmia, atrioventricular block or concomitant neuromuscular adverse events are poor prognostic factors that may lead to IIST.^9 11^ Thus, in our study, the two main indications for IIST were the presence of other irAEs and an unfavorable hemodynamic outcome. Nevertheless, the risk of sudden death related to ventricular arrhythmia should also be considered because this complication can occur even in the presence of a stable hemodynamic state.^6^ A recent work has shown that the persistence of elevated troponin T (≥1.5 ng/mL) at hospital discharge was associated with a fourfold increased risk of major adverse cardiac events.^11^ In our work, the persistence of elevated troponin or its increase was also a frequent indication of IIST.

As soon as the evolution is unfavorable under corticosteroids, the guidelines recommend IIST, but this is an empirical strategy due to lack of evidence.^13 14^ The pathophysiology of ICI-induced myocarditis remains poorly understood.^23^ Since the histological lesions observed are similar to those observed during acute cardiac transplant cell rejection, experts logically recommended drugs indicated in this situation. These include high doses of methylprednisolone (1 g per day for 3 days) as well as ATG, MMF or tacrolimus. However, an in vitro study showed that B cells also widely express both PD-1 and PD-L1. Thus, the blocking of PD-1/PD-L1 can restore and enhance B-cell proliferation, interleukin-6 production and antibodies to self-antigens, such as acetylcholine receptor, striated muscle or Ro proteins.^24^ This may justify other therapies, such as plasma exchange, intravenous Ig, infliximab (antitumor necrosis factor-alpha (TNF-α)), rituximab (anti-CD20), tocilizumab (anti-interleukin-6), alemtuzumab (anti-CD52) and abatacept (CTLA-4 agonist).

In our study, these strategies were used either alone or in combination. Although our work was not designed to determine which of them is the most effective, it does suggest a deleterious effect of infliximab, which was associated with a significant increase in the risk of cardiovascular death. This monoclonal antibody is indicated in the treatment of several chronic inflammatory diseases because the neutralization of TNF-α regulates the inflammatory response by reducing the release of interleukin-1, interleukin-6 and TNF-β. Although TNF-α is increased in the serum, macrophages and myocardial cells of patients with heart failure syndrome,^25^ cardiac toxicity has been well reported with infliximab, and it has been shown to adversely affect the clinical condition of patients with moderate to severe heart failure.^26 27^ In addition, cases of myocarditis have also been described under this treatment.^28^ These data, combined with those from our study, suggest that infliximab should not be used as first-line therapy after corticosteroids in patients with ICI-induced myocarditis.

Our work has many limitations that we acknowledge. This study was retrospective in design, and we pooled patients from our center with patients in previously published case reports. Thus, the quality of the data collected in case reports was not controlled. However, we chose to limit the number of case reports by including only those where patients received IIST. Moreover, data from patients from our center were prospectively collected into a controlled and protected database immediately on admission. We acknowledge that this methodology is questionable, but it made it possible to increase the number of patients treated with IIST for this infrequent serious disease. Due to the design, we were unable to determine the exact rate of patients requiring IIST in the whole population. Nevertheless, in the subgroup of patients admitted to our center, this rate was 14%. Several relevant covariates could not be analyzed, such as the evolution of troponin and natriuretic peptide levels, doses of drugs administered, and the evolution of cancer after the ICI-induced myocarditis episode. Mortality rates should be interpreted with caution, as follow-up in case reports was most often limited to a very short period following the adverse event. Finally, results on the potential deleterious effect of infliximab should be interpreted with caution in regard to the small sample size and the lack of controlled design. These are only exploratory data, but they raise concern over infliximab use as second-line therapy.

## Conclusion

The need for IIST was more common in patients who developed myocarditis very early after the start of ICI therapy, especially combination of ICIs, as well as when hemodynamic/electrical instability or neuromuscular irAEs occurred. In patients receiving IIST therapy, treatment with infliximab was associated with a significantly increased risk of cardiovascular death. Thus, this study identifies patients at high risk of adverse events and provides opportunities for further work to determine the most effective immunosuppressive therapeutic strategy for ICI-induced myocarditis.

10.1136/jitc-2020-001887.supp2 Supplementary data
