# Supplementary material for: Intensified immunosuppressive therapy in patients with immune checkpoint inhibitor-induced myocarditis
Source: J Immunother Cancer. 2020 Dec 8;8(2):e001887. doi: 10.1136/jitc-2020-001887 (PMC7725077; doi:10.1136/jitc-2020-001887)
Supplement: Supplementary data [file jitc-2020-001887supp001.pdf]

Additional file 1: Table S1. References of case reports identified in PubMed

1. Arangalage D, Delyon J, Lermuzeaux M, et al. Survival After Fulminant Myocarditis Induced by Immune-Checkpoint Inhibitors. *Annals of Internal Medicine*. 2017;167(9):683-684. doi:10.7326/L17-0396

2. Xing Q, Zhang Z-W, Lin Q-H, et al. Myositis-myasthenia gravis overlap syndrome complicated with myasthenia crisis and myocarditis associated with anti-programmed cell death-1 (sintilimab) therapy for lung adenocarcinoma. *Ann Transl Med*. 2020;8(5):250. doi:10.21037/atm.2020.01.79

3. Tay RY, Blackley E, McLean C, et al. Successful use of equine anti-thymocyte globulin (ATGAM) for fulminant myocarditis secondary to nivolumab therapy. *Br J Cancer*. 2017;117(7):921-924. doi:10.1038/bjc.2017.253

4. Frigeri M, Meyer P, Banfi C, et al. Immune Checkpoint Inhibitor-Associated Myocarditis: A New Challenge for Cardiologists. *Canadian Journal of Cardiology*. 2018;34(1):92.e1-92.e3. doi:10.1016/j.cjca.2017.09.025

5. Kimura T, Fukushima S, Miyashita A, et al. Myasthenic crisis and polymyositis induced by one dose of nivolumab. *Cancer Sci*. 2016;107(7):1055-1058. doi:10.1111/cas.12961

6. Balanescu DV, Donisan T, Palaskas N, et al. Immunomodulatory treatment of immune checkpoint inhibitor-induced myocarditis: Pathway toward precision-based therapy. *Cardiovascular Pathology*. 2020;47:107211. doi:10.1016/j.carpath.2020.107211

7. Chen W. Acute fatal myocarditis after a single dose of anti-PD-1 immunotherapy, autopsy findings: a case report. *Cardiovascular Pathology*. 2020;46. doi:10.1016/j.carpath.2020.107202

8. Agrawal N, Khunger A, Vachhani P, et al. Cardiac Toxicity Associated with Immune Checkpoint Inhibitors: Case Series and Review of the Literature. *CRO*. 2019;12(1):260-276. doi:10.1159/000498985

9. Fazel M, Jedlowski PM. Severe Myositis, Myocarditis, and Myasthenia Gravis with Elevated Anti-Striated Muscle Antibody following Single Dose of Ipilimumab-Nivolumab Therapy in a Patient with Metastatic Melanoma. Case Reports in Immunology. doi:https://doi.org/10.1155/2019/2539493

10. Chen Q, Huang D-S, Zhang L-W, Li Y-Q, Wang H-W, Liu H. Fatal myocarditis and rhabdomyolysis induced by nivolumab during the treatment of type B3 thymoma. *Clinical Toxicology*. 2018;56(7):667-671. doi:10.1080/15563650.2017.1401079

11. Saibil SD, Bonilla L, Majeed H, et al. Fatal myocarditis and rhabdomyositis in a patient with stage IV melanoma treated with combined ipilimumab and nivolumab. *Curr Oncol*. 2019;26(3):e418-e421. doi:10.3747/co.26.4381

12. Ansari-Gilani K, Tirumani SH, Smith DA, et al. Myocarditis associated with immune checkpoint inhibitor therapy: a case report of three patients. *Emerg Radiol*. 2020;27(4):455-460. doi:10.1007/s10140-020-01765-6

13. Gallegos C, Rottmann D, Nguyen VQ, Baldassarre LA. Myocarditis with checkpoint inhibitor immunotherapy: case report of late gadolinium enhancement on cardiac magnetic resonance with pathology correlate. *Eur Heart J Case Rep*. 2019;3(1). doi:10.1093/ehjcr/yty149

14. Guo CW, Alexander M, Dib Y, et al. A closer look at immune-mediated myocarditis in the era of combined checkpoint blockade and targeted therapies. *European Journal of Cancer*. 2020;124:15-24. doi:10.1016/j.ejca.2019.09.009

15. Mahmood SS, Chen CL, Shapnik N, Krishnan U, Singh HS, Makker V. Myocarditis with tremelimumab plus durvalumab combination therapy for endometrial cancer: A case report. *Gynecol Oncol Rep*. 2018;25:74-77. doi:10.1016/j.gore.2018.05.014

16. McDowall LM, Fernando SL, Ange N, Yun J, Chia KKM. Immune checkpoint inhibitor-mediated myocarditis and ventricular tachycardia storm. *HeartRhythm Case Rep*. 2019;5(10):497-500. doi:10.1016/j.hrcr.2019.06.006

17. Wang H, Tian R, Gao P, Wang Q, Zhang L. Tocilizumab for Fulminant Programmed Death 1 Inhibitor–Associated Myocarditis. *Journal of Thoracic Oncology*. 2020;15(3):e31-e32. doi:10.1016/j.jtho.2019.09.080

18. Jain V, Mohebtash M, Rodrigo ME, Ruiz G, Atkins MB, Barac A. Autoimmune Myocarditis Caused by Immune Checkpoint Inhibitors Treated With Antithymocyte Globulin. *Journal of Immunotherapy*. 2018;41(7):332–335. doi:10.1097/CJI.0000000000000239

19. Norwood TG, Westbrook BC, Johnson DB, et al. Smoldering myocarditis following immune checkpoint blockade. *J Immunother Cancer*. 2017;5(1):91. doi:10.1186/s40425-017-0296-4

20. Rota E, Varese P, Agosti S, et al. Concomitant myasthenia gravis, myositis, myocarditis and polyneuropathy, induced by immune-checkpoint inhibitors: A life-threatening continuum of neuromuscular and cardiac toxicity. *eNeurologicalSci*. 2019;14:4-5. doi:10.1016/j.ensci.2018.11.023

21. Johnson DB, Balko JM, Compton ML, et al. Fulminant Myocarditis with Combination Immune Checkpoint Blockade. *New England Journal of Medicine*. 2016;375(18):1749-1755. doi:10.1056/NEJMoa1609214

22. Salem J-E, Allenbach Y, Vozy A, et al. Abatacept for Severe Immune Checkpoint Inhibitor-Associated Myocarditis. *N Engl J Med*. 2019;380(24):2377-2379. doi:10.1056/NEJMc1901677

23. Esfahani K, Buhlaiga N, Thébault P, Lapointe R, Johnson NA, Miller WH. Alemtuzumab for Immune-Related Myocarditis Due to PD-1 Therapy. *New England Journal of Medicine*. 2019;380(24):2375-2376. doi:10.1056/NEJMc1903064

24. Shah M, Tayar JH, Abdel-Wahab N, Suarez-Almazor ME. Myositis as an adverse event of immune checkpoint blockade for cancer therapy. *Semin Arthritis Rheum*. 2019;48(4):736-740. doi:10.1016/j.semarthrit.2018.05.006
