# Supplementary material for: Intensified immunosuppressive therapy in patients with immune checkpoint inhibitor-induced myocarditis
Source: J Immunother Cancer. 2020 Dec 8;8(2):e001887. doi: 10.1136/jitc-2020-001887 (PMC7725077; doi:10.1136/jitc-2020-001887)
Supplement: Supplementary data [file jitc-2020-001887supp002.pdf]

Intensified immunosuppressive therapy (IIST) in patients with immune checkpoint inhibitor-induced myocarditis

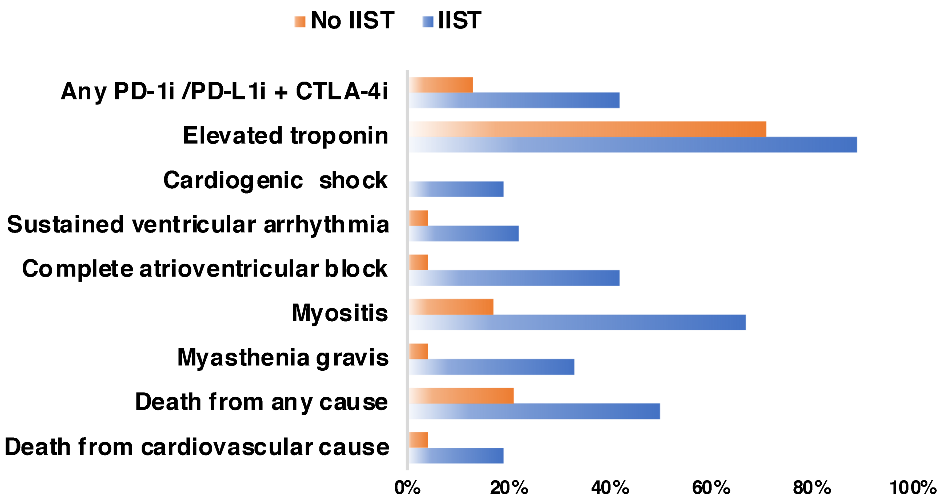

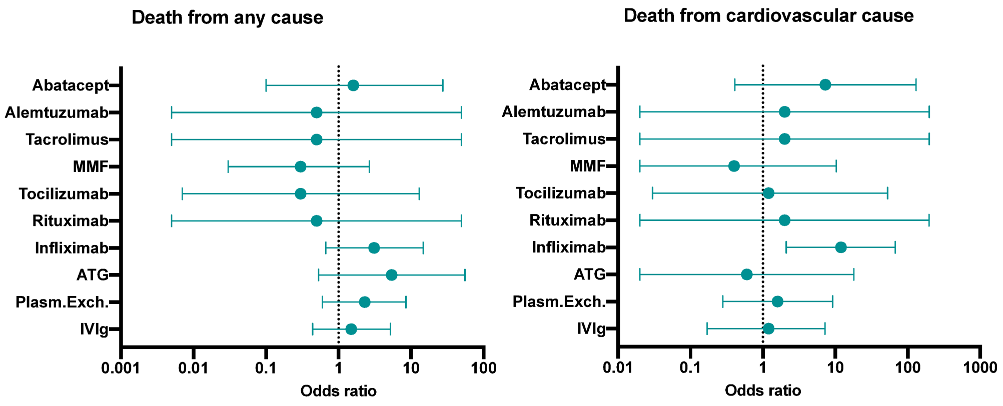

Authors

Jennifer Cautela, Sarah Zeriouh, Melanie Gaubert, Laurent Bonello, Marc Laine, Michael Peyrol, Franck Paganelli, Nathalie Lalevee, Fabrice Barlesi, Franck Thuny

Correspondence

franck.thuny@gmail.com from The Mediterranean University Centre of Cardio-Oncology, Aix Marseille University, France

In Brief

The need for IIST was more common in patients who developed immune checkpoint inhibitor-induced (ICI) myocarditis very early after the start of ICI therapy, as well as when hemodynamic/electrical instability or neuromuscular adverse events occurred. Treatment with infliximab might be associated with an increased risk of cardiovascular death.
